# Activity of the Novel Aminomethylcycline KBP-7072 and Comparators against 1,057 Geographically Diverse Recent Clinical Isolates from the SENTRY Surveillance Program, 2019

**DOI:** 10.1128/AAC.01397-21

**Published:** 2022-01-18

**Authors:** Michael D. Huband, Jennifer D. Thompson, Nabina D. Gurung, Qingmei Liu, Li Li, Jay Zhang, Jennifer M. Streit, Mariana Castanheira

**Affiliations:** a JMI Laboratories, North Liberty, Iowa, USA; b KBP Biosciences Co., Ltd., Jinan, China; c KBP Biosciences USA, Inc., Princeton, New Jersey, USA

**Keywords:** KBP-7072, aminomethylcycline, antibacterial, tetracyclines

## Abstract

KBP-7072 is a novel broad-spectrum tetracycline (aminomethylcycline) antibacterial in clinical development (oral and intravenous formulations) for the treatment of acute bacterial skin and skin structure infections, community-acquired bacterial pneumonia, and complicated intra-abdominal infections. KBP-7072 is active against many of the World Health Organization priority pathogens. In this study, KBP-7072 and tetracycline class comparators were susceptibility tested against 1,057 geographically diverse surveillance isolates from 2019 according to Clinical and Laboratory Standards Institute (CLSI) guidelines. KBP-7072 demonstrated potent *in vitro* activity against Gram-positive and Gram-negative bacterial pathogens. KBP-7072 was active against Staphylococcus aureus (MIC_50/90_, 0.06/0.12 mg/liter), methicillin-resistant S. aureus (MIC_50/90_, 0.06/0.12 mg/liter), *S. lugdunensis* (MIC_50/90_, 0.03/0.03 mg/liter), and other coagulase-negative staphylococci (MIC_50/90_, 0.06/0.25 mg/liter). KBP-7072 was active against Enterococcus faecalis (MIC_50/90_, 0.03/0.06 mg/liter) and vancomycin-susceptible and -nonsusceptible E. faecium (MIC_50/90_, 0.03/0.03 mg/liter); Streptococcus pneumoniae (MIC_50/90_, ≤0.015/0.03 mg/liter), including penicillin- and tetracycline-resistant strains; S. agalactiae (MIC_50/90_, 0.03/0.06 mg/liter), including macrolide-resistant strains; S. pyogenes (MIC_50/90_, 0.03/0.03 mg/liter); and viridans group streptococci, including *S. anginosus* group (MIC_50/90_, ≤0.015/0.03 mg/liter) isolates. KBP-7072 inhibited 90.2% (MIC_50/90_, 0.25/2 mg/liter) of all *Enterobacterales* isolates, including expanded-spectrum β-lactamase-phenotype strains at ≤2 mg/liter. KBP-7072 demonstrated potent activity against Acinetobacter baumannii*-calcoaceticus* species complex and Stenotrophomonas maltophilia isolates (MIC_50/90_ values, 0.5/1 mg/liter), Haemophilus influenzae (MIC_50/90_, 0.12/0.25 mg/liter; 100.0% inhibited at ≤0.25 mg/liter), and Moraxella catarrhalis (MIC_50/90_, 0.06/0.06 mg/liter). Based on MIC_90_ values, KBP-7072 *in vitro* activity was generally superior to that the other tetracycline class comparators tested. The potent activity of KBP-7072, including resistant organism groups, merits further clinical investigation in infections where these organisms are likely to occur.

## INTRODUCTION

The effectiveness of tetracycline antibacterials has declined since their initial discovery and introduction in the late 1940s, primarily due to the emergence of resistance caused by efflux and ribosomal protection mechanisms (1, 2). This resistance has diminished the effectiveness of both narrow-spectrum (e.g., tetracycline) and expanded-spectrum (e.g., doxycycline and minocycline) tetracyclines against medically important Gram-positive and Gram-negative bacteria (1–4). Tigecycline represents a broad-spectrum intravenous tetracycline (glycylcycline subclass) that overcomes these common bacterial efflux and ribosome protection mechanisms (3). Eravacycline is a fully synthetic third-generation intravenous tetracycline belonging to the fluorocycline subclass. KBP-7072 and omadacycline (aminomethylcycline subclass) are recent third-generation tetracycline antibacterials with a broad spectrum of activity, intravenous and oral dosing options, and activity against isolates expressing common efflux and ribosome protection mechanisms (5–9).

**FIG 1:**
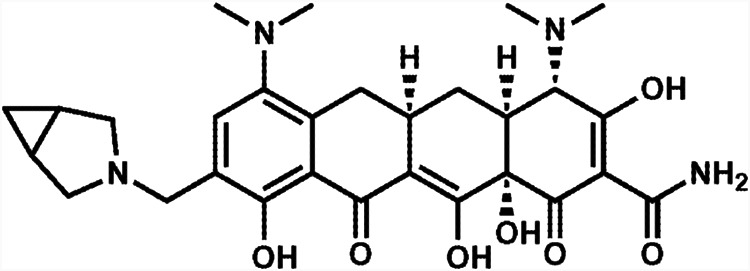
Chemical structure of KBP-7072.

KBP-7072 (Fig. 1) has completed phase I clinical development (10–13). KBP-7072 is in phase II clinical development (oral and intravenous formulations) for the treatment of acute bacterial skin and skin structure infection (ABSSSI), community-acquired bacterial pneumonia (CAP), and complicated intra-abdominal infection (cIAI).

KBP-7072 exhibits potent *in vitro* antibacterial activity against many of the organisms listed on the World Health Organization (WHO) priority pathogen list, including methicillin-resistant Staphylococcus aureus, penicillin-nonsusceptible Streptococcus pneumoniae, vancomycin-resistant Enterococcus faecium, ampicillin-resistant Haemophilus influenzae, and carbapenem-resistant Acinetobacter baumannii (6, 7, 14). In the present study, we evaluated the *in vitro* antimicrobial activity of KBP-7072 and comparator agents against 1,057 isolates of Gram-positive cocci and Gram-negative bacilli collected in 2019 from 117 medical centers located in 35 countries as a part of the SENTRY Antimicrobial Surveillance Program. When available, evaluations of resistant organism subsets were also included for specific pathogen groups.

## RESULTS

### Overall activity of KBP-7072.

Cumulative percent inhibition and MIC_50_/MIC_90_ data for KBP-7072 against 1,057 recent geographically diverse organisms and organism groups are detailed in Table 1. KBP-7072 and comparator agent susceptibility data, including MIC range, MIC_50/90_, percent susceptible (S), percent intermediate (I), and percent resistant (R) according to CLSI or FDA breakpoint interpretive criteria, are presented in Tables 2 and 3. Based on MIC_90_ values, KBP-7072 was the most active agent tested against S. aureus, including methicillin-resistant S. aureus (MRSA) (MIC_90_, 0.12 mg/liter), tetracycline-resistant S. aureus (MIC_90_, 0.25 mg/liter), and enterococci (MIC_90_, 0.03 to 0.06 mg/liter), including vancomycin-nonsusceptible strains (Tables 1 and 2). KBP-7072 demonstrated potent *in vitro* activity against streptococci, inhibiting 100.0% of Streptococcus anginosus (MIC_90_, 0.03 mg/liter), S. agalactiae (MIC_90_, 0.06 mg/liter), S. pyogenes (MIC_90_, 0.03 mg/liter), and S. pneumoniae (MIC_90_, 0.03 mg/liter), including penicillin-resistant, tetracycline-resistant, and macrolide-resistant strains at ≤0.12 mg/liter (Table 1). Against 410 *Enterobacterales* isolates, KBP-7072 (MIC_90_, 2 mg/liter) was comparable in activity to tigecycline (MIC_90_, 2 mg/liter), 8-fold more active than omadacycline and minocycline (MIC_90_, 16 mg/liter), 16-fold more active than doxycycline (MIC_90_, 32 mg/liter), and >32-fold more active than tetracycline (MIC_90_, >64 mg/liter) (Table 3). When tested against A. baumannii isolates, KBP-7072 (MIC_90_, 1 mg/liter) was 4-fold more active than tigecycline (MIC_90_, 4 mg/liter), 8-fold more active than omadacycline and minocycline (MIC_90_, 8 mg/liter), >32-fold more active than doxycycline (MIC_90_, >32 mg/liter), and >64-fold more active than tetracycline (MIC_90_, >64 mg/liter) (Table 3). All H. influenzae isolates (100.0%) were inhibited by ≤0.25 mg/liter KBP-7072 (Table 3).

**TABLE 1:** Antimicrobial activity of KBP-7072 against 1,057 geographically diverse, recent clinical isolates

Organism/organism group (no. of isolates)	No. and cumulative % of isolates inhibited at MIC (mg/liter) of^*a*^:													MIC_50_ (mg/liter)	MIC_90_ (mg/liter)
	≤0.015	0.03	0.06	0.12	0.25	0.5	1	2	4	8	16	32	>32		
Staphylococcus aureus (104)	0, 0.0	17, 16.3	75, 88.5	9, 97.1	2, 99.0	1, 100.0								0.06	0.12
Methicillin-susceptible (52)	0, 0.0	7, 13.5	39, 88.5	6, 100.0										0.06	0.12
Methicillin-resistant (52)	0, 0.0	10, 19.2	36, 88.5	3, 94.2	2, 98.1	1, 100.0								0.06	0.12
Tetracycline-resistant (12)	0, 0.0	3, 25.0	7, 83.3	0, 83.3	1, 91.7	1, 100.0								0.06	0.25
*S. lugdunensis* (20)	9, 45.0	11, 100.0												0.03	0.03
Other coagulase-negative staphylococci (22)	0, 0.0	7, 31.8	6, 59.1	3, 72.7	4, 90.9	2, 100.0								0.06	0.25
Enterococcus faecalis (51)	0, 0.0	38, 74.5	13, 100.0											0.03	0.06
E. faecium (50)	12, 24.0	35, 94.0	2, 98.0	1, 100.0										0.03	0.03
Vancomycin-nonsusceptible (MIC, ≥8 mg/liter) (24)	8, 33.3	15, 95.8	0, 95.8	1, 100.0										0.03	0.03
Streptococcus pneumoniae (127)	89, 70.1	37, 99.2	1, 100.0											≤0.015	0.03
Penicillin-susceptible oral (MIC, ≤0.06 mg/liter) (76)	55, 72.4	20, 98.7	1, 100.0											≤0.015	0.03
Penicillin-intermediate oral (MIC, 0.12–1 mg/liter) (27)	20, 74.1	7, 100.0												≤0.015	0.03
Penicillin-resistant oral (MIC, ≥2 mg/liter) (24)	14, 58.3	10, 100.0												≤0.015	0.03
Tetracycline-resistant (MIC, ≥4 mg/liter) (35)	23, 65.7	12, 100.0												≤0.015	0.03
S. agalactiae (52)	0, 0.0	46, 88.5	6, 100.0											0.03	0.06
Macrolide-resistant (erythromycin MIC, ≥1 mg/liter) (22)	0, 0.0	19, 86.4	3, 100.0											0.03	0.06
S. pyogenes (51)	4, 7.8	44, 94.1	3, 100.0											0.03	0.03
*S. anginosus* group (17)	12, 70.6	5, 100.0												≤0.015	0.03
*Enterobacterales* (410)		0, 0.0	2, 0.5	65, 16.3	173, 58.5	78, 77.6	35, 86.1	17, 90.2	31, 97.8	7, 99.5	2, 100.0			0.25	2
* Enterobacterales* (133) tetracycline-resistant			0, 0.0	8, 6.0	16, 18.0	30, 40.6	26, 60.2	14, 70.7	30, 93.2	7, 98.5	2, 100.0			1	4
Citrobacter freundii species complex (22)			0, 0.0	2, 9.1	12, 63.6	6, 90.9	2, 100.0							0.25	0.5
* C. koseri* (21)			0, 0.0	12, 57.1	7, 90.5	2, 100.0								0.12	0.25
Enterobacter cloacae species complex (50)				0, 0.0	33, 66.0	14, 94.0	1, 96.0	0, 96.0	2, 100.0					0.25	0.5
Ceftazidime-susceptible (MIC, ≤4 mg/liter) (32)				0, 0.0	19, 59.4	11, 93.8	1, 96.9	0, 96.9	1, 100.0					0.25	0.5
Ceftazidime-nonsusceptible (MIC, ≥8 mg/liter) (18)				0, 0.0	14, 77.8	3, 94.4	0, 94.4	0, 94.4	1, 100.0					0.25	0.5
Escherichia coli (77)		0, 0.0	2, 2.6	40, 54.5	20, 80.5	12, 96.1	2, 98.7	1, 100.0						0.12	0.5
Non-ESBL phenotype (51)		0, 0.0	2, 3.9	34, 70.6	13, 96.1	2, 100.0								0.12	0.25
ESBL phenotype (26)			0, 0.0	6, 23.1	7, 50.0	10, 88.5	2, 96.2	1, 100.0						0.25	1
Klebsiella aerogenes (21)				0, 0.0	18, 85.7	1, 90.5	1, 95.2	0, 95.2	1, 100.0					0.25	0.5
K. oxytoca (53)			0, 0.0	7, 13.2	39, 86.8	3, 92.5	1, 94.3	3, 100.0						0.25	0.5
K. pneumoniae (80)			0, 0.0	4, 5.0	44, 60.0	14, 77.5	10, 90.0	5, 96.2	3, 100.0					0.25	1
Non-ESBL phenotype (53)			0, 0.0	4, 7.5	35, 73.6	6, 84.9	4, 92.5	3, 98.1	1, 100.0					0.25	1
ESBL phenotype (27)				0, 0.0	9, 33.3	8, 63.0	6, 85.2	2, 92.6	2, 100.0					0.5	2
Morganella morganii (20)					0, 0.0	6, 30.0	9, 75.0	4, 95.0	0, 95.0	1, 100.0				1	2
Proteus mirabilis (22)						0, 0.0	1, 4.5	1, 9.1	14, 72.7	5, 95.5	1, 100.0			4	8
*Providencia* spp. (22)					0, 0.0	2, 9.1	5, 31.8	3, 45.5	11, 95.5	1, 100.0				4	4
Serratia marcescens (22)					0, 0.0	18, 81.8	3, 95.5	0, 95.5	0, 95.5	0, 95.5	1, 100.0			0.5	1
Acinetobacter baumannii*-calcoaceticus* species complex (22)	0, 0.0	3, 13.6	5, 36.4	1, 40.9	1, 45.5	6, 72.7	6, 100.0							0.5	1
Carbapenem (meropenem) resistant (MIC, ≥8) (13)				0, 0.0	1, 7.7	6, 53.8	6, 100.0							0.5	1
Pseudomonas aeruginosa (22)					0, 0.0	1, 4.5	0, 4.5	1, 9.1	5, 31.8	11, 81.8	3, 95.5	0, 95.5	1, 100.0	8	16
Stenotrophomonas maltophilia (22)				0, 0.0	4, 18.2	14, 81.8	3, 95.5	0, 95.5	0, 95.5	1, 100.0				0.5	1
Haemophilus influenzae (52)		0, 0.0	2, 3.8	41, 82.7	9, 100.0									0.12	0.25
*H. parainfluenzae* (12)		0, 0.0	1, 8.3	0, 8.3	9, 83.3	2, 100.0								0.25	0.5
Moraxella catarrhalis (21)	1, 4.8	2, 14.3	18, 100.0											0.06	0.06

a Greater than the highest concentration tested.

**TABLE 2:** Activity of KBP-7072 and tetracycline class comparators against Gram-positive clinical isolates

Antimicrobial agent (no. of isolates)	MIC_50_ (mg/liter)	MIC_90_ (mg/liter)	MIC range (mg/liter)	CLSI^*a*^		
				%S	%I	%R
Staphylococcus aureus (104)						
KBP-7072	0.06	0.12	0.03–0.5			
Doxycycline	0.12	1	0.03–8	96.2	3.8	0.0
Minocycline	0.06	0.25	0.03–16	96.2	1.9	1.9
Omadacycline	0.12	0.25	0.06–4	97.1,^*b*^^,^^*c*^ 97.1^*d*^^,^^*e*^	0.0, 0.0	2.9, 2.9
Tetracycline	0.25	16	0.12–64	86.5	1.9	11.5
Tigecycline	0.12	0.25	0.03–1	99.0^*c*^		
S. aureus (52) methicillin susceptible						
KBP-7072	0.06	0.12	0.03–0.12			
Doxycycline	0.06	0.25	0.03–1	100.0	0.0	0.0
Minocycline	0.06	0.25	0.03–0.25	100.0	0.0	0.0
Omadacycline	0.12	0.25	0.06–0.25	100.0,^*b*^^,^^*c*^ 100.0^*d*^^,^^*e*^	0.0, 0.0	0.0, 0.0
Tetracycline	0.25	0.5	0.12–16	94.2	1.9	3.8
Tigecycline	0.12	0.12	0.03–0.25	100.0^*c*^		
S. aureus (52) methicillin resistant						
KBP-7072	0.06	0.12	0.03–0.5			
Doxycycline	0.12	2	0.06–8	92.3	7.7	0.0
Minocycline	0.06	0.5	0.03–16	92.3	3.8	3.8
Omadacycline	0.12	0.25	0.06–4	94.2,^*b*^^,^^*c*^ 94.2^*d*^^,^^*e*^	0.0, 0.0	5.8, 5.8
Tetracycline	0.25	32	0.12–64	78.8	1.9	19.2
Tigecycline	0.12	0.25	0.06–1	98.1^*c*^		
S. aureus (12) tetracycline resistant						
KBP-7072	0.06	0.25	0.03–0.5			
Doxycycline	2	8	1–8	66.7	33.3	0.0
Minocycline	0.25	16	0.06–16	66.7	15.6	15.6
Omadacycline	0.12	2	0.06–4	83.3,^*b*^^,^^*c*^ 83.3^*d*^^,^^*e*^	0.0, 0.0	16.7, 16.7
Tetracycline	32	64	16–64	0.0	0.0	100.0
Tigecycline	0.12	0.5	0.12–1	91.7		8.3
*S. lugdunensis* (20)						
KBP-7072	0.03	0.03	≤0.015–0.03			
Doxycycline	0.06	0.06	0.015–4	100.0	0.0	0.0
Minocycline	0.03	0.03	≤0.015–0.12	100.0	0.0	0.0
Omadacycline	0.06	0.06	≤0.015–0.06	100.0^*b*^^,^^*c*^	0.0	0.0
Tetracycline	0.12	0.12	≤0.03–64	95.0	0.0	5.0
Tigecycline	0.03	0.06	0.015–0.12			
Other coagulase-negative staphylococci^*f*^ (22)						
KBP-7072	0.06	0.25	0.03–0.5			
Doxycycline	0.12	8	0.06–16	86.4	4.5	9.1
Minocycline	0.12	0.5	0.03–1	100.0	0.0	0.0
Omadacycline	0.12	1	0.06–1			
Tetracycline	0.25	64	0.12 to >64	81.8	0.0	18.2
Tigecycline	0.06	0.25	0.03–0.5			
Enterococcus faecalis (51)						
KBP-7072	0.03	0.06	0.03–0.06			
Doxycycline	8	8	0.12–32	45.1	49.0	5.9
Minocycline	8	16	0.06–16	33.3	35.3	31.4
Omadacycline	0.12	0.12	0.06–0.25	100.0^*b*^^,^^*c*^	0.0	0.0
Tetracycline	32	64	0.25 to >64	31.4	0.0	68.6
Tigecycline	0.12	0.12	0.06–0.25	100.0^*g*^		
E. faecium (50)						
KBP-7072	0.03	0.03	≤0.015–0.12			
Doxycycline	0.12	8	0.06–8	76.0	24.0	0.0
Minocycline	0.06	16	0.03–16	70.0	10.0	20.0
Omadacycline	0.06	0.12	0.03–0.5			
Tetracycline	0.5	>64	0.12 to >64	54.0	0.0	46.0
Tigecycline	0.06	0.06	0.03–0.25			
E. faecium (24) vancomycin nonsusceptible (MIC, ≥32 mg/liter)						
KBP-7072	0.03	0.03	≤0.015–0.12			
Doxycycline	0.12	8	0.06–8	75.0	25.0	0.0
Minocycline	0.06	16	0.03–16	66.7	12.5	20.8
Omadacycline	0.06	0.12	0.03–0.5			
Tetracycline	0.25	>64	0.12 to >64	50.0	0.0	50.0
Tigecycline	0.06	0.06	0.03–0.25			
Streptococcus pneumoniae (127)						
KBP-7072	≤0.015	0.03	≤0.015–0.06			
Doxycycline	0.12	8	0.03–16	70.9	2.4	26.8
Minocycline	0.12	8	0.03–32			
Omadacycline	0.03	0.06	≤0.015–0.12	100.0^*b*^^,^^*c*^	0.0	0.0
Tetracycline	0.25	64	0.12–64	72.4	0.0	27.6
Tigecycline	0.03	0.03	0.015–0.06	100.0^*c*^		
S. pneumoniae (55) erythromycin resistant (MIC, ≥1 mg/liter)						
KBP-7072	≤0.015	0.03	≤0.015–0.03			
Doxycycline	4	16	0.03–16	38.2	3.6	58.2
Minocycline	4	8	0.03–32			
Omadacycline	0.03	0.06	≤0.015–0.12	100.0^*b*^^,^^*c*^	0.0	0.0
Tetracycline	32	64	0.12–64	40.0	0.0	60.0
Tigecycline	0.03	0.03	0.015–0.06	100.0^*c*^		
S. pneumoniae (24) penicillin resistant (MIC, ≥2 mg/liter) oral						
KBP-7072	≤0.015	0.03	≤0.015–0.03			
Doxycycline	2	8	0.03–16	41.7	0.0	58.3
Minocycline	4	8	0.03–16			
Omadacycline	0.06	0.06	≤0.015–0.12	100.0^*b*^^,^^*c*^	0.0	0.0
Tetracycline	16	64	0.12–64	41.7	0.0	58.3
Tigecycline	0.03	0.03	0.015–0.06	100.0^*c*^		
S. pneumoniae (35) tetracycline resistant (MIC, ≥2 mg/liter)						
KBP-7072	≤0.015	0.03	≤0.015–0.03			
Doxycycline	8	16	0.5–16	0.0	2.9	97.1
Minocycline	8	16	0.5–32			
Omadacycline	0.06	0.06	0.03–0.12	100.0^*b*^^,^^*c*^	0.0	0.0
Tetracycline	32	64	4–64	0.0	0.0	100.0
Tigecycline	0.03	0.03	0.015–0.06	100.0^*c*^		
S. agalactiae (52)						
KBP-7072	0.03	0.06	0.03–0.06			
Doxycycline	8	16	0.12–16			
Minocycline	16	32	0.06–32			
Omadacycline	0.12	0.25	0.06–0.25			
Tetracycline	32	64	0.25–64	19.2	0.0	80.8
Tigecycline	0.06	0.06	0.03–0.06	100.0^*c*^		
S. agalactiae (22) macrolide resistant (erythromycin MIC, ≥1 mg/liter)						
KBP-7072	0.03	0.06	0.03–0.06			
Doxycycline	16	16	0.12–16			
Minocycline	16	32	0.06–32			
Omadacycline	0.12	0.25	0.06–0.25			
Tetracycline	32	64	0.25–64	4.5	0.0	95.5
Tigecycline	0.06	0.06	0.03–0.06	100.0^*c*^		
S. pyogenes (51)						
KBP-7072	0.03	0.03	≤0.015–0.06			
Doxycycline	0.12	8	0.06–16			
Minocycline	0.12	8	0.06–16			
Omadacycline	0.06	0.12	0.03–0.25	98.0^*b*^^,^^*c*^	2.0	0.0
Tetracycline	0.25	32	0.06–64	80.4	0.0	19.6
Tigecycline	0.03	0.06	0.015–0.12	100.0^*c*^		
*S. anginosus* group (17)						
KBP-7072	≤0.015	0.03	≤0.015–0.03			
Doxycycline	0.25	8	0.06–16			
Minocycline	0.06	16	≤0.015–16			
Omadacycline	0.06	0.12	≤0.015–0.12	100.0^*b*^^,^^*c*^	0.0	0.0
Tetracycline	0.5	32	0.06–64	70.6	5.9	23.5
Tigecycline	0.015	0.03	≤0.008–0.06	100.0^*c*^		

a Criteria as published by CLSI (22).

b Using ABSSSI breakpoints.

c FDA breakpoints.

d Using CABP breakpoints.

e FDA breakpoints for MSSA only (applied for all S. aureus).

f Organisms include Staphylococcus capitis (2), S. epidermidis (11), *S. haemolyticus* (3), S. hominis (1), *S. pettenkoferi* (1), S. pseudintermedius*/intermedius/delphini* (3), and *S. simulans* (1).

g FDA breakpoints applied to all E. faecalis but approved for vancomycin-susceptible isolates only.

**TABLE 3:** Activity of KBP-7072 and tetracycline class comparators against Gram-negative clinical isolates

Antimicrobial agent (no. of isolates)	MIC_50_ (mg/liter)	MIC_90_ (mg/liter)	MIC range (mg/liter)	CLSI^*a*^		
				%S	%I	%R
*Enterobacterales* (410)						
KBP-7072	0.25	2	0.06–16			
Doxycycline	2	32	0.5 to >32	67.6	5.6	26.8
Minocycline	2	16	0.25 to >32	79.0	6.1	14.9
Omadacycline	2	16	0.5 to >32			
Tetracycline	2	>64	0.5 to >64	64.4	3.2	32.4
Tigecycline	0.5	2	0.12–8	93.4^*b*^	5.6	1.0
*Enterobacterales* (133)^*c*^ tetracycline resistant						
KBP-7072	1	4	0.12–16			
Doxycycline	32	>32	4 to >32	3.0	15.0	82.0
Minocycline	8	>32	1 to >32	38.3	15.8	45.9
Omadacycline	4	32	0.5 to >32			
Tetracycline	>64	>64	16 to >64	0.0	0.0	100
Tigecycline	1	4	0.12–8	79.7	17.3	3.0
Citrobacter freundii species complex (22)						
KBP-7072	0.25	0.5	0.12–1			
Doxycycline	2	8	1–32	86.4	4.5	9.1
Minocycline	1	8	0.5–16	86.4	9.1	4.5
Omadacycline	1	4	1–8			
Tetracycline	1	8	0.5 to >64	86.4	4.5	9.1
Tigecycline	0.25	1	0.12–2	100.0^*b*^	0.0	0.0
*C. koseri* (21)						
KBP-7072	0.12	0.25	0.12–0.5			
Doxycycline	1	2	0.5–4	100.0	0.0	0.0
Minocycline	0.5	2	0.5–4	100.0	0.0	0.0
Omadacycline	1	1	0.5–2			
Tetracycline	1	2	1–4	100.0	0.0	0.0
Tigecycline	0.25	0.25	0.12–0.5	100.0^*b*^	0.0	0.0
Enterobacter cloacae species complex (50)						
KBP-7072	0.25	0.5	0.25–4			
Doxycycline	2	2	1–16	92.0	0.0	8.0
Minocycline	2	4	0.5–32	96.0	0.0	4.0
Omadacycline	2	4	1–16	94.0^*b*^^,^^*d*^	2.0	4.0
Tetracycline	2	4	1 to >64	90.0	0.0	10.0
Tigecycline	0.5	0.5	0.25–4	96.0^*b*^	4.0	0.0
E. cloacae species complex (18) ceftazidime-nonsusceptible (MIC, ≥8 mg/liter)						
KBP-7072	0.25	0.5	0.25–4			
Doxycycline	2	4	1–16	94.4	0.0	5.6
Minocycline	2	4	0.5–32	94.4	0.0	5.6
Omadacycline	2	4	1–16	94.4^*b*^^,^^*d*^	0.0	5.6
Tetracycline	2	16	1 to >64	88.9	0.0	11.1
Tigecycline	0.5	0.5	0.25–4	94.4^*b*^	5.6	0.0
Escherichia coli (77)						
KBP-7072	0.12	0.5	0.06–2			
Doxycycline	2	32	0.5 to >32	58.4	14.3	27.3
Minocycline	1	8	0.5–32	84.4	7.8	7.8
Omadacycline	1	2	0.5–16			
Tetracycline	2	>64	0.5 to >64	57.1	0.0	42.9
Tigecycline	0.25	0.5	0.12–4	98.7^*b*^	1.3	0.0
E. coli (26) ESBL phenotype						
KBP-7072	0.25	1	0.12–2			
Doxycycline	8	32	1–32	38.5	26.9	34.6
Minocycline	2	8	0.5–32	84.6	7.7	7.7
Omadacycline	2	4	0.5–16			
Tetracycline	>64	>64	1 to >64	38.5	0.0	61.5
Tigecycline	0.25	1	0.12–4	96.2^*b*^	3.8	0.0
Klebsiella aerogenes (21)						
KBP-7072	0.25	0.5	0.25–4			
Doxycycline	2	4	0.5 to >32	90.5	0.0	9.5
Minocycline	2	2	1 to >32	95.2	0.0	4.8
Omadacycline	2	2	1–32			
Tetracycline	2	8	1 to >64	85.7	4.8	9.5
Tigecycline	0.5	0.5	0.25–8	95.2^*b*^	0.0	4.8
K. oxytoca (53)						
KBP-7072	0.25	0.5	0.12–2			
Doxycycline	1	8	0.5–32	88.7	3.8	7.5
Minocycline	1	4	0.25–16	94.3	1.9	3.8
Omadacycline	1	2	0.5–16			
Tetracycline	1	8	0.5 to >64	88.7	1.9	9.4
Tigecycline	0.25	1	0.12–2	100.0^*b*^	0.0	0.0
K. pneumoniae (80)						
KBP-7072	0.25	1	0.12–4			
Doxycycline	1	16	0.5 to >32	71.2	3.8	25.0
Minocycline	2	8	0.5 to >32	83.8	7.5	8.8
Omadacycline	2	8	0.5–32	87.5^*b*^^,^^*d*^^,^^*e*^	7.5	5.0
Tetracycline	2	>64	0.5 to >64	72.5	0.0	27.5
Tigecycline	0.5	1	0.25–4	96.2^*b*^	3.8	0.0
K. pneumoniae (27) ESBL phenotype						
KBP-7072	0.5	2	0.25–4			
Doxycycline	8	32	1 to >32	40.7	11.1	48.1
Minocycline	4	>32	1 to >32	74.1	7.4	18.5
Omadacycline	4	16	1–32	81.5^*b*^^,^^*d*^^,^^*e*^	7.4	11.1
Tetracycline	>64	>64	1 to >64	40.7	0.0	59.3
Tigecycline	0.5	2	0.25–4	92.6^*b*^	7.4	0.0
Morganella morganii (20)						
KBP-7072	1	2	0.5–8			
Doxycycline	32	>32	1 to >32	45.0	0.0	55.0
Minocycline	4	32	1 to >32	50.0	5.0	45.0
Omadacycline	8	8	2 to >32			
Tetracycline	32	>64	1 to >64	45.0	0.0	55.0
Tigecycline	1	2	0.5–4	95.0^*b*^	5.0	0.0
Proteus mirabilis (22)						
KBP-7072	4	8	1–16			
Doxycycline	32	>32	16 to >32	0.0	0.0	100.0
Minocycline	16	32	8 to >32	0.0	13.6	86.4
Omadacycline	16	>32	8 to >32			
Tetracycline	32	64	32–64	0.0	0.0	100.0
Tigecycline	4	4	1–8	31.8^*b*^	59.1	9.1
*Providencia* spp. (22)^*f*^						
KBP-7072	4	4	0.5–8			
Doxycycline	>32	>32	2 to >32	4.5	4.5	90.9
Minocycline	16	>32	2 to >32	18.2	22.7	59.1
Omadacycline	32	32	4 to >32			
Tetracycline	64	>64	2 to >64	9.1	0.0	90.9
Tigecycline	2	4	0.5–4	86.4^*b*^	13.6	0.0
Serratia marcescens (22)						
KBP-7072	0.5	1	0.5–16			
Doxycycline	4	16	2–32	59.1	22.7	18.2
Minocycline	2	4	2–32	90.9	4.5	4.5
Omadacycline	4	8	2 to >32			
Tetracycline	8	>64	4 to >64	4.5	45.5	50.0
Tigecycline	1	2	0.5–8	95.5^*b*^	0.0	4.5
Acinetobacter baumannii*-calcoaceticus* species complex (22)						
KBP-7072	0.5	1	0.03–1			
Doxycycline	1	>32	0.06 to >32	63.6	9.1	27.3
Minocycline	0.5	8	0.03–16	72.7	18.2	9.1
Omadacycline	2	8	0.12–8			
Tetracycline	16	>64	1 to >64	40.9	4.5	54.5
Tigecycline	2	4	0.12–4			
A. baumannii*-calcoaceticus* species complex (13) carbapenem resistant						
KBP-7072	0.5	1	0.25–1			
Doxycycline	8	>32	0.25 to >32	38.5	15.3	46.2
Minocycline	4	16	0.25–16	53.8	30.8	15.4
Omadacycline	4	8	2–8			
Tetracycline	64	>64	4 to >64	7.7	0.0	92.3
Tigecycline	4	4	2–4			
Pseudomonas aeruginosa (22)						
KBP-7072	8	16	0.5 to >32			
Doxycycline	16	32	4 to >32			
Minocycline	16	32	4 to >32			
Omadacycline	32	>32	2 to >32			
Tetracycline	16	32	4 to >64			
Tigecycline	8	16	0.5 to >16			
Stenotrophomonas maltophilia (22)						
KBP-7072	0.5	1	0.25–8			
Doxycycline	2	4	1–16			
Minocycline	0.5	1	0.25–8	95.5	4.5	0.0
Omadacycline	4	8	2–32			
Tetracycline	32	64	16–64			
Tigecycline	1	2	0.5–16			
Haemophilus influenzae (52)						
KBP-7072	0.12	0.25	0.06–0.5			
Doxycycline	0.5	0.5	0.12–1			
Minocycline	0.25	0.5	0.12–0.5			
Omadacycline	0.5	1	0.25–2	100.0^*b*^^,^^*e*^	0.0	0.0
Tetracycline	0.5	0.5	0.25–8	98.1	0.0	1.9
Tigecycline	0.25	0.25	0.06–0.5	94.2		
*H. parainfluenzae* (12)						
KBP-7072	0.25	0.5	0.06–0.5			
Doxycycline	0.5	2	0.25–16			
Minocycline	1	2	0.25–4			
Omadacycline	1	2	0.25–2	100.0^*b*^^,^^*e*^	0.0	0.0
Tetracycline	0.5	1	0.25–32	91.7	0.0	8.3
Tigecycline	0.5	0.5	0.06–1			
Moraxella catarrhalis (21)						
KBP-7072	0.06	0.06	≤0.015 to 0.06			
Doxycycline	0.12	0.12	0.06–0.25			
Minocycline	0.06	0.06	0.03–0.06			
Omadacycline	0.12	0.25	0.06–0.25			
Tetracycline	0.25	0.5	0.12–0.5	100.0	0.0	0.0
Tigecycline	0.06	0.06	0.03–0.06			

a Criteria as published by CLSI (22).

b FDA breakpoints.

c Organisms include Citrobacter freundii species complex (2), E. cloacae species complex (5), E. coli (33), K. aerogenes (2), K. oxytoca (5), K. pneumoniae (22), M. morganii (11), P. mirabilis (22), *P. rettgeri* (11), P. stuartii (9), and S. marcescens (11).

d Using ABSSSI breakpoints.

e Using CABP breakpoints.

f Organisms include Providencia alcalifaciens (1), *P. rettgeri* (12), and P. stuartii (9).

### Activity of KBP-7072 against staphylococci.

KBP-7072 (MIC_50/90_, 0.06/0.12 mg/liter; 100.0% inhibited at ≤0.5 mg/liter) demonstrated potent *in vitro* activity against 104 S. aureus isolates, including methicillin-susceptible S. aureus (MSSA) (MIC_50/90_, 0.06/0.12 mg/liter; 100.0% inhibited at ≤0.12 mg/liter) and MRSA (MIC_50/90_, 0.06/0.12 mg/liter; 100.0% inhibited at ≤0.5 mg/liter) organism subsets (Tables 1 and 2). S. aureus resistance to tetracycline was 11.5% (Table 2). Based on S. aureus MIC_90_ values, KBP-7072 (MIC_90_, 0.12 mg/liter) was 2-fold more active than minocycline (MIC_90_, 0.25 mg/liter; 96.2% susceptible), omadacycline (MIC_90_, 0.25 mg/liter; 97.1% susceptible), and tigecycline (MIC_90_, 0.25 mg/liter; 99.0% susceptible), 8-fold more active than doxycycline (MIC_90_, 1 mg/liter; 96.2% susceptible), and 128-fold more active than tetracycline (MIC_90_, 16 mg/liter; 86.5% susceptible) (Table 2).

KBP-7072 (MIC_50/90_, 0.03/0.03 mg/liter; 100.0% inhibited at ≤0.03 mg/liter) demonstrated potent *in vitro* activity against 20 Staphylococcus lugdunensis isolates where resistance to tetracycline was 5.0% (Table 2). Based on *S. lugdunensis* MIC_90_ values, KBP-7072 (MIC_90_, 0.03 mg/liter) was comparable in activity to minocycline (MIC_90_, 0.03 mg/liter; 100.0% susceptible), 2-fold more active than doxycycline (MIC_90_, 0.06 mg/liter; 100.0% susceptible), omadacycline (MIC_90_, 0.06 mg/liter; 100.0% susceptible), and tigecycline (MIC_90_, 0.06 mg/liter) and 4-fold more active than tetracycline (MIC_90_, 0.12 mg/liter; 95.0% susceptible) (Tables 1 and 2).

KBP-7072 (MIC_50/90_, 0.06/0.25 mg/liter, 100% inhibited at ≤0.5 mg/liter) and tigecycline (MIC_50/90_, 0.06/0.25 mg/liter) were comparable in activity against 22 other coagulase-negative staphylococci (CoNS) isolates (Table 2). Resistance to doxycycline and tetracycline was 9.1% and 18.2%, respectively (Table 2). Based on MIC_90_ values, KBP-7072 (MIC_90_, 0.25 mg/liter) was 2-fold more active than minocycline (MIC_90_, 0.5 mg/liter; 100.0% susceptible), 4-fold more active than omadacycline (MIC_90_, 1 mg/liter), 32-fold more active than doxycycline (MIC_90_, 8 mg/liter; 86.4% susceptible), and 256-fold more active than tetracycline (MIC_90_, 64 mg/liter; 81.8% susceptible) against other CoNS isolates (Table 2).

### Activity of KBP-7072 against enterococci.

KBP-7072 was highly active against 51 E. faecalis (MIC_50/90_, 0.03/0.06 mg/liter; 100.0% inhibited at ≤0.06 mg/liter) isolates (Table 1). Based on E. faecalis MIC_90_ values, KBP-7072 (MIC_90_, 0.06 mg/liter) was 2-fold more active than omadacycline and tigecycline (MIC_90_ values, 0.12 mg/liter; 100.0% susceptible) (Table 2). Doxycycline (MIC_90_, 8 mg/liter; 45.1% susceptible), minocycline (MIC_90_, 16 mg/liter; 33.3% susceptible), and tetracycline (MIC_90_, 64 mg/liter; 31.4% susceptible) demonstrated limited activity against E. faecalis isolates (Table 2).

KBP-7072 was the most active antibacterial tested against 50 E. faecium (MIC_50/90_, 0.03/0.03 mg/liter; 100.0% inhibited at ≤0.12 mg/liter) isolates, and its activity was not adversely affected by susceptibility or nonsusceptibility to vancomycin (Tables 1 and 2). Based on E. faecium MIC_90_ values, KBP-7072 (MIC_90_, 0.03 mg/liter) was 2-fold more active than tigecycline (MIC_90_, 0.06 mg/liter) and 4-fold more active than omadacycline (MIC_90_, 0.12 mg/liter) (Table 2). Doxycycline (MIC_90,_ 8 mg/liter; 76.0% susceptible), minocycline (MIC_90_, 16 mg/liter; 70.0% susceptible), and tetracycline (MIC_90_, >64 mg/liter; 54.0% susceptible) demonstrated reduced activity against E. faecium isolates (Table 2).

### Activity of KBP-7072 against streptococci.

S. pneumoniae isolates (*n* = 127) were highly susceptible to KBP-7072 (MIC_90_, 0.03 mg/liter; 100.0% inhibited at ≤0.06 mg/liter), and its activity was not adversely affected by resistance to erythromycin (MIC_90_, 0.03 mg/liter), penicillin (MIC_90_, 0.03 mg/liter), or tetracycline (MIC_90_, 0.03 mg/liter) (Tables 1 and 2). Based on S. pneumoniae MIC_90_ values, KBP-7072 (MIC_90_, 0.03 mg/liter) was comparable in activity to tigecycline (MIC_90_, 0.03 mg/liter; 100.0% susceptible), 2-fold more active than omadacycline (MIC_90_, 0.06 mg/liter; 100.0% susceptible), 256-fold more active than doxycycline (MIC_90_, 8 mg/liter; 70.9% susceptible) and minocycline (MIC_90,_ 8 mg/liter), and 2,048-fold more active than tetracycline (MIC_90_, 64 mg/liter; 72.4% susceptible) (Table 2). Resistance to erythromycin, penicillin, and tetracycline among S. pneumoniae isolates was 43.3%, 18.9% (oral breakpoint), and 27.6%, respectively (Table 2). Resistance of S. pneumoniae isolates to other commonly used antibacterials was 43.3% for azithromycin, 7.1% for ceftriaxone (oral breakpoints), 22.0% for clindamycin, and 18.1% for trimethoprim-sulfamethoxazole (data not shown).

Beta-hemolytic streptococci, including 52 S. agalactiae (MIC_50/90_, 0.03/0.06 mg/liter) and 51 S. pyogenes isolates (MIC_50/90_, 0.03/0.03 mg/liter), were inhibited by ≤0.06 mg/liter of KBP-7072 regardless of erythromycin (macrolide) resistance (Table 1). Based on S. agalactiae MIC_90_ values, KBP-7072 (MIC_90_, 0.06 mg/liter) was comparable in activity to tigecycline (MIC_90_, 0.06 mg/liter; 100.0% susceptible), 4-fold more active than omadacycline (MIC_90_, 0.25 mg/liter), 256-fold more active than doxycycline (MIC_90_, 16 mg/liter), 512-fold more active than minocycline (MIC_90_, 32 mg/liter), and 1,024-fold more active than tetracycline (MIC_90_, 64 mg/liter; 19.2% susceptible) (Table 2). Similarly, against 51 S. pyogenes isolates, KBP-7072 (MIC_90_, 0.03 mg/liter) was 2-fold more active than tigecycline (MIC_90_, 0.06 mg/liter; 100.0% susceptible), 4-fold more active than omadacycline (MIC_90_, 0.12 mg/liter; 98.0% susceptible), 256-fold more active than doxycycline and minocycline (MIC_90_ values, 8 mg/liter), and 1,024-fold more active than tetracycline (MIC_90_, 32 mg/liter; 80.4% susceptible) (Table 2).

All Streptococcus anginosus group isolates (*n* = 17) were inhibited by low concentrations of KBP-7072 (MIC_90_, 0.03 mg/liter; 100.0% inhibited at ≤0.03 mg/liter) and tigecycline (MIC_90_, 0.03 mg/liter; 100.0% susceptible) (Table 2). Based on MIC_90_ values, KBP-7072 was 4-fold more active than omadacycline (MIC_90_, 0.12 mg/liter; 100.0% susceptible), 256-fold more active than doxycycline (MIC_90_ 8 mg/liter), 512-fold more active than minocycline (MIC_90_, 16 mg/liter), and 1,024-fold more active than tetracycline (MIC_90_, 32 mg/liter; 70.6% susceptible) (Table 2). Resistance of *S. anginosus* group isolates to other commonly used antibacterials were 29.4% for azithromycin, 11.8% for clindamycin, and 29.4% for erythromycin (data not shown).

### Activity of KBP-7072 against *Enterobacterales* isolates.

KBP-7072 (MIC_50/90_, 0.25/2 mg/liter; 90.2% inhibited at ≤2 mg/liter) and tigecycline (MIC_50/90_, 0.5/2 mg/liter; 93.4% susceptible) were the most active tetracycline class compounds tested against 410 *Enterobacterales* isolates (Table 3). Based on MIC_90_ values, KBP-7072 (MIC_90_, 2 mg/liter) was 8-fold more active than omadacycline (MIC_90_, 16 mg/liter) and minocycline (MIC_90_, 16 mg/liter; 79.0% susceptible), 16-fold more active than doxycycline (MIC_90_ 32 mg/liter; 67.6% susceptible), and >32-fold more active than tetracycline (MIC_90_, >64 mg/liter; 64.4% susceptible) against *Enterobacterales* isolates (Table 3). KBP-7072 (MIC_50/90_, 1/4 mg/liter) was comparable in activity to tigecycline against 133 tetracycline-resistant *Enterobacterales* isolates (Table 3). Comparator agents with activity of >90.0% against *Enterobacterales* isolates included amikacin (MIC_90_, 4 mg/liter; 98.5% susceptible) and meropenem (MIC_90_, 0.06 mg/liter; 97.8% susceptible) (data not shown).

### Activity of KBP-7072 against *Citrobacter* species isolates.

KBP-7072 (MIC_90_, 0.5 mg/liter; 100.0% inhibited at ≤1 mg/liter) and tigecycline (MIC_90_, 1 mg/liter; 100.0% susceptible) were the most active tetracycline class agents tested against 22 Citrobacter freundii species complex isolates (Table 3). Based on C. freundii species complex MIC_90_ values, KBP-7072 (MIC_90_, 0.5 mg/liter) was 8-fold more active than omadacycline (MIC_90_, 4 mg/liter) and 16-fold more active than doxycycline, minocycline, and tetracycline (MIC_90_, 8 mg/liter; 86.4% susceptible) (Table 3).

All Citrobacter koseri isolates (*n* = 21) were susceptible (100.0%) to doxycycline, minocycline, tetracycline, and tigecycline. KBP-7072 and tigecycline were the most active tetracycline class agents against *C. koseri*, with MIC_90_ values of 0.25 mg/liter.

### Activity of KBP-7072 against Enterobacter cloacae species complex isolates.

KBP-7072 (MIC_90_, 0.5 mg/liter; 100.0% inhibited at ≤4 mg/liter) and tigecycline (MIC_50/90_, 0.5/0.5 mg/liter; 96.0% susceptible) were the most active tetracycline class agents tested against 50 Enterobacter cloacae species complex isolates (Tables 1 and 3). Based on E. cloacae species complex MIC_90_ values, KBP-7072 (MIC_90_, 0.5 mg/liter) was 4-fold more active than doxycycline (MIC_90_, 2 mg/liter; 92.0% susceptible) and 8-fold more active than minocycline (MIC_90_, 4 mg/liter; 96.0% susceptible), omadacycline (MIC_90_, 4 mg/liter; 94.0% susceptible), and tetracycline (MIC_90_, 4 mg/liter; 90.0% susceptible) (Table 3). KBP-7072 (MIC_50/90_, 0.25/0.5 mg/liter) was equally active against ceftazidime-susceptible and ceftazidime-nonsusceptible (AmpC-derepressed phenotype) E. cloacae species complex isolates (Tables 1 and 3).

### Activity of KBP-7072 against Escherichia coli isolates.

KBP-7072 (MIC_50/90_, 0.12/0.5 mg/liter; 100.0% inhibited at ≤2 mg/liter) was active against 77 Escherichia coli isolates, including a subset of 26 expanded-spectrum β-lactamase (ESBL)-phenotype E. coli isolates (MIC_50/90_, 0.25/1 mg/liter; 100.0% inhibited at ≤2 mg/liter) (Tables 1 and 3). Against ESBL-phenotype E. coli isolates, KBP-7072 (MIC_90_, 1 mg/liter) was comparable in activity to tigecycline (MIC_90_, 1 mg/liter; 96.2% susceptible), 4-fold more active than omadacycline (MIC_90_, 4 mg/liter), 8-fold more active than minocycline (MIC_90_, 8 mg/liter; 84.6% susceptible), 32-fold more active than doxycycline (MIC_90_, 32 mg/liter; 38.5% susceptible), and >64-fold more active than tetracycline (MIC_90_, >64 mg/liter; 38.5% susceptible) (Table 3).

### Activity of KBP-7072 against Klebsiella species isolates.

KBP-7072 (MIC_90_, 0.5 mg/liter; 100.0% inhibited at ≤4 mg/liter) and tigecycline (MIC_90_, 0.5 mg/liter; 95.2% susceptible) were the most active tetracycline class agents tested against 21 Klebsiella aerogenes isolates (Table 3). Based on K. aerogenes MIC_90_ values, KBP-7072 (MIC_90_, 0.5 mg/liter) was 4-fold more active than minocycline (MIC_90_, 2 mg/liter; 95.2% susceptible) and omadacycline (MIC_90_, 2 mg/liter), 8-fold more active than doxycycline (MIC_90_, 4 mg/liter; 90.5% susceptible), and 16-fold more active than tetracycline (MIC_90_, 8 mg/liter; 85.7% susceptible) (Table 3).

KBP-7072 (MIC_90_, 0.5 mg/liter; 100.0% inhibited at ≤2 mg/liter) and tigecycline (MIC_90_, 1 mg/liter; 100.0% susceptible) were comparable in activity against 53 K. oxytoca isolates (Table 3). Based on K. oxytoca MIC_90_ values, KBP-7072 (MIC_90_, 0.5 mg/liter) was 4-fold more active than omadacycline (MIC_90_, 2 mg/liter), 8-fold more active than minocycline (MIC_90_, 4 mg/liter; 94.3% susceptible), and 16-fold more active than doxycycline and tetracycline (MIC_90_ values, 8 mg/liter; 88.7% susceptible) (Table 3).

KBP-7072 (MIC_50/90_, 0.25/1 mg/liter; 100.0% inhibited at ≤4 mg/liter) was active against 80 K. pneumoniae isolates, including a subset of 27 ESBL-phenotype K. pneumoniae isolates (MIC_50/90_, 0.5/2 mg/liter; 100.0% inhibited at ≤4 mg/liter) (Tables 1 and 3). Against ESBL-phenotype K. pneumoniae isolates, KBP-7072 (MIC_90_, 2 mg/liter) was comparable in activity to tigecycline (MIC_90_, 2 mg/liter; 92.6% susceptible), 8-fold more active than omadacycline (MIC_90_, 16 mg/liter; 81.5% susceptible), 16-fold more active than doxycycline (MIC_90_, 32 mg/liter; 40.7% susceptible), >16-fold more active than minocycline (MIC_90_, >32 mg/liter; 74.1% susceptible), and >32-fold more active than tetracycline (MIC_90_, >64 mg/liter; 40.7% susceptible) (Table 3).

### Activity of KBP-7072 against Morganella morganii isolates.

KBP-7072 (MIC_50/90_, 1/2 mg/liter; 95.0% inhibited at ≤2 mg/liter) and tigecycline (MIC_50/90_, 1/2 mg/liter; 95.0% susceptible) were the most active tetracycline class agents tested against 20 Morganella morganii isolates (Tables 1 and 3). Reduced activity was observed for other tetracycline class agents, including omadacycline (MIC_90_, 8 mg/liter), minocycline (MIC_90_, 32 mg/liter; 50.0% susceptible), doxycycline (MIC_90_, >32 mg/liter; 45.0% susceptible), and tetracycline (MIC_90_, >64 mg/liter; 45.0% susceptible) (Table 3).

### Activity of KBP-7072 against Proteus mirabilis isolates.

All tetracycline class agents demonstrated reduced or limited activity against 22 Proteus mirabilis isolates, including KBP-7072 (MIC_90_, 8 mg/liter; 72.7% inhibited at ≤4 mg/liter), doxycycline (MIC_90_, >32 mg/liter; 0.0% susceptible), minocycline (MIC_90_, 32 mg/liter; 0.0% susceptible), omadacycline (MIC_90_, >32 mg/liter), tetracycline (MIC_90_, 64 mg/liter; 0.0% susceptible), and tigecycline (MIC_90_, 4 mg/liter; 31.8% susceptible) (Tables 1 and 3).

### Activity of KBP-7072 against *Providencia* species isolates.

KBP-7072 (MIC_90_, 4 mg/liter; 95.5% inhibited at ≤4 mg/liter) and tigecycline (MIC_90_, 4 mg/liter; 86.4% susceptible) were the most active tetracycline class agents tested against 22 *Providencia* species isolates (Tables 1 and 3). Other tetracycline class agents demonstrated limited activity against *Providencia* species isolates, including doxycycline (MIC_90_, >32 mg/liter; 4.5% susceptible), minocycline (MIC_90_, >32 mg/liter; 18.2% susceptible), omadacycline (MIC_90_, 32 mg/liter), and tetracycline (MIC_90_, >64 mg/liter; 9.1% susceptible) (Table 3).

### Activity of KBP-7072 against Serratia marcescens isolates.

KBP-7072 (MIC_90_, 1 mg/liter; 95.5% inhibited at ≤1 mg/liter) and tigecycline (MIC_90_, 2 mg/liter; 95.5% susceptible) were the most active tetracycline class agents tested against 22 Serratia marcescens isolates (Tables 1 and 3). Based on S. marcescens MIC_90_ values, KBP-7072 (MIC_90_, 1 mg/liter) was 4-fold more active than minocycline (MIC_90_, 4 mg/liter; 90.9% susceptible), 8-fold more active than omadacycline (MIC_90_, 8 mg/liter), 16-fold more active than doxycycline (MIC_90_, 16 mg/liter; 59.1% susceptible), and >64-fold more active than tetracycline (MIC_90_, >64 mg/liter; 4.5% susceptible) (Table 3).

### Activity of KBP-7072 against nonfermenters.

Based on *in vitro* activity, KBP-7072 (MIC_50/90_, 0.5/1 mg/liter; 100.0% inhibited at ≤1 mg/liter) was the most potent tetracycline class agent tested against 22 A. baumannii *calcoaceticus* species complex isolates (Table 3). KBP-7072 (MIC_90_, 1 mg/liter) was 4-fold more active than tigecycline (MIC_90_, 4 mg/liter), 8-fold more active than minocycline (MIC_90_, 8 mg/liter, 72.7% susceptible) and omadacycline (MIC_90_, 8 mg/liter), >32-fold more active than doxycycline (MIC_90_, >32 mg/liter; 63.6% susceptible), and >64-fold more active than tetracycline (MIC_90_, >64 mg/liter; 40.9% susceptible) (Table 3). Comparator agent susceptibilities against A. baumannii *calcoaceticus* species complex isolates ranged from 36.4% for piperacillin-tazobactam to 59.1% for amikacin (data not shown).

The activity of KBP-7072 (MIC_50/90_, 8/16 mg/liter) and all tetracycline class agents was limited against 22 Pseudomonas aeruginosa isolates (Table 3).

KBP-7072 (MIC_50/90_, 0.5/1 mg/liter) and minocycline (MIC_50/90_, 0.5/1 mg/liter; 95.5% susceptible) demonstrated potent *in vitro* activity against 22 Stenotrophomonas maltophilia isolates (Table 3). Based on S. maltophilia MIC_90_ values, KBP-7072 (MIC_90_, 1 mg/liter) was 2-fold more active than tigecycline (MIC_90_, 2 mg/liter), 8-fold more active than omadacycline (MIC_90_, 8 mg/liter), and 64-fold more active than tetracycline (Table 3). Susceptibility of S. maltophilia isolates to other commonly used antibacterials was 22.7% for ceftazidime, 77.3% for levofloxacin, and 90.5% for trimethoprim-sulfamethoxazole (data not shown).

### Activity of KBP-7072 against fastidious organism groups.

All tetracycline class agents were very active against 52 H. influenzae isolates, including KBP-7072 (MIC_50/90_, 0.12/0.25 mg/liter; 100.0% inhibited at ≤0.25 mg/liter), doxycycline (MIC_50/90_, 0.5/0.5 mg/liter), minocycline (MIC_50/90_, 0.25/0.5 mg/liter), omadacycline (MIC_50/90_, 0.5/1 mg/liter; 100.0% susceptible), tetracycline (MIC_50/90_, 0.5/0.5 mg/liter, 98.1% susceptible), and tigecycline (MIC_50/90_, 0.25/0.25 mg/liter; 94.2% susceptible) (Table 3).

KBP-7072 (MIC_50/90_, 0.25/0.5 mg/liter; 100.0% inhibited at ≤0.5 mg/liter) was active against 12 *H. parainfluenzae* isolates (Tables 1 and 3). Based on *H. parainfluenzae* MIC_90_ values, KBP-7072 (MIC_90_, 0.5 mg/liter) was comparable in activity to tigecycline (MIC_90_, 0.5 mg/liter), 2-fold more active than tetracycline (MIC_90,_ 1 mg/liter; 91.7% susceptible), and 4-fold more active than doxycycline (MIC_90_, 2 mg/liter), minocycline (MIC_90_, 2 mg/liter), and omadacycline (MIC_90_, 2 mg/liter; 100.0% susceptible) (Table 3).

All tetracycline class agents, including KBP-7072 (MIC_50/90_, 0.06/0.06 mg/liter; 100.0% inhibited at ≤0.06 mg/liter), were active against 21 Moraxella catarrhalis isolates (Tables 1 and 3).

## DISCUSSION

The WHO has defined which resistant organism groups should be prioritized to help guide the discovery and development of new antibacterial agents (14). Of these, carbapenem-resistant A. baumannii and *Enterobacteriaceae* were identified as priority 1 (critical) pathogens, vancomycin-resistant E. faecium and MRSA were identified as priority 2 (high) pathogens, and ampicillin-resistant H. influenzae and penicillin-nonsusceptible S. pneumoniae were identified as priority 3 (medium) pathogens (14). The WHO also stressed the importance of orally active agents for the treatment of ESBL-producing *Enterobacteriaceae* (14). Few therapeutic options are available for some infections, such as those caused by carbapenem-resistant A. baumannii and multidrug-resistant organisms (15–17).

KBP-7072 has demonstrated dose-proportional pharmacokinetic/pharmacodynamic (PK/PD) properties in both animal models and phase I clinical studies supporting once-daily oral or intravenous administration (10–13, 18–20). Specifically, PK/PD evaluation of KBP-7072 against S. aureus, and S. pneumoniae was investigated in the neutropenic murine pneumonia model and KBP-7072 human PK data were obtained from a phase 1 oral dosing study (10, 13, 18, 20). In a 10-day multiple ascending dose study using healthy volunteers, the therapeutic dose of KBP-7072 was determined likely to be less than 200 mg/day (20). The PK/PD and probability of target attainment (PTA) analysis indicated that KBP-7072 would be efficacious for Gram-positive pathogens at a dose level of 50 mg and for Gram negatives (A. baumannii) at a dose level of 200 mg. The therapeutic dose for KBP-7072 is projected to be lower than the current daily oral dose for another aminomethylcycline class antibacterial (omadacycline) in community-acquired bacterial pneumonia and acute bacterial skin and skin structure infection (21). A lower overall KBP-7072 therapeutic dose is supported by the lower MIC_90_ values for KBP-7072 compared to omadacycline against key organism groups, including MRSA (2-fold); tetracycline-resistant S. aureus (8-fold); E. faecium (4-fold); S. pneumoniae (2-fold), including penicillin-nonsusceptible strains; beta-hemolytic streptococci (4-fold); *Enterobacterales* (8-fold), including ESBL-phenotype and tetracycline-resistant strains; A. baumannii (8-fold); S. maltophilia (8-fold); and H. influenzae (4-fold).

The *in vitro* activity of KBP-7072 was unaffected by isolates displaying resistance to tetracycline. KBP-7072 remained active against isolates displaying resistance to other antibacterial agents, including ampicillin, ceftazidime, erythromycin, penicillin, and vancomycin.

The potent *in vitro* activity of KBP-7072 against A. baumannii is also supported by a prior study using 531 isolates that included carbapenem-resistant, colistin-resistant, ESBL-positive, metallo-β-lactamase-producing, and tetracycline-resistant strains (6).

In summary, KBP-7072 demonstrated potent *in vitro* activity against a collection of 1,057 recent geographically diverse clinical isolates, including staphylococci, streptococci, enterococci, *Enterobacteriaceae*, H. influenzae, A. baumannii, S. maltophilia, and drug-resistant organisms and organism groups. This study supports the continued clinical development of KBP-7072 in serious infections, including those caused by drug-resistant organisms.

## MATERIALS AND METHODS

### Organisms.

Geographically diverse, recent (2019) bacterial clinical isolates (*n* = 1,057) were collected from 117 medical centers located in 35 countries, including the United States (56 medical centers; 372 isolates; 35.2% overall), Europe (19 countries, 36 medical centers; 375 isolates; 35.5% overall), Latin America (6 countries, 10 medical centers; 151 isolates; 14.3% overall), and the Asia-Pacific region (9 countries, 15 medical centers; 159 isolates; 15.0% overall) as part of the SENTRY Surveillance Program. The surveillance isolates utilized in this study were randomly selected from patients with skin and skin structure infections (589 isolates; 55.7% overall), pneumonia in hospitalized patients (281 isolates; 26.6% overall), and community-acquired respiratory tract infections (187 isolates; 17.7% overall) and included only 1 isolate/patient/infection episode. The percentage of tetracycline-resistant isolates in this study generally mimicked the 2019 worldwide SENTRY Surveillance Program distributions for A. baumannii (54.5% versus 60.3% in SENTRY), *Enterobacterales* (32.4% versus 34.6% in SENTRY), H. influenzae (1.9% versus 1.4% in SENTRY), S. aureus (11.5% versus 6.0% in SENTRY), S. agalactiae (80.8% versus 79.6% in SENTRY), S. pneumoniae (27.6% versus 24.7% in SENTRY), and S. pyogenes (19.6% versus 21.2% in SENTRY). Organism identifications were confirmed by matrix-assisted laser desorption ionization time-of-flight mass spectroscopy (Bruker Daltonics, Bremen, Germany).

### Compounds.

KBP-7072 and omadacycline (KBP-3039) powders were supplied by KBP Biosciences Co., Ltd. (Jinan, China). Doxycycline, tetracycline, and tigecycline powders were obtained from the United States Pharmacopeial Convention (Rockville, MD, USA). Minocycline powder was obtained from Sigma-Aldrich (Millipore, Saint Louis, MO, USA).

### Resistance phenotype definitions.

CLSI interpretive criteria were applied to define the extended-spectrum β-lactamase (ESBL) phenotype for E. coli and K. pneumoniae isolates, MRSA, vancomycin-susceptible and -nonsusceptible E. faecium, penicillin-susceptible, -intermediate, -resistant (oral breakpoints), and tetracycline-resistant S. pneumoniae, erythromycin (macrolide)-resistant S. agalactiae and S. pyogenes, ceftazidime-susceptible and -nonsusceptible E. cloacae, carbapenem (meropenem)-resistant A. baumannii, and ampicillin-resistant H. influenzae (22). Most ESBL-phenotype isolates were subjected to molecular characterization using next-generation sequencing and high-resolution *in silico* analysis (16).

### Antimicrobial susceptibility testing.

Broth microdilution susceptibility testing was conducted at JMI Laboratories according to Clinical and Laboratory Standards Institute M07 (23) and M100 (22) guidelines. Results were interpreted using CLSI breakpoint criteria for doxycycline, minocycline, and tetracycline (22) and FDA breakpoint criteria for omadacycline and tigecycline (24). Freshly prepared cation-adjusted Mueller-Hinton broth was used to inoculate the MIC panels. Doxycycline, minocycline, tetracycline, tigecycline, and meropenem were used as bridge compounds as needed to the historical susceptibility data from the SENTRY Antimicrobial Surveillance Program.

### Quality control.

JMI Laboratories followed current CLSI quality assurance practices when performing susceptibility tests. MIC values were validated by concurrently testing the CLSI-recommended (22) American Type Culture Collection (ATCC) quality control strains. Tested quality control strains included E. faecalis ATCC 29212, E. coli ATCC 25922, E. coli ATCC 35218, P. aeruginosa ATCC 27853, and S. aureus ATCC 29213. All (100.0%) of the doxycycline (18/18), minocycline (15/15), omadacycline (18/18), tetracycline (25/25), and tigecycline (18/18) MIC values obtained were within CLSI-approved quality control ranges (22). The inoculum density during susceptibility testing was monitored by bacterial colony counts.
